# Isolation and Characterization of Nano-Hydroxyapatite from Salmon Fish Bone

**DOI:** 10.3390/ma8085253

**Published:** 2015-08-21

**Authors:** Jayachandran Venkatesan, Baboucarr Lowe, Panchanathan Manivasagan, Kyong-Hwa Kang, Elna P. Chalisserry, Sukumaran Anil, Dong Gyu Kim, Se-Kwon Kim

**Affiliations:** 1 Marine Bioprocess Research Center, Pukyong National University, Busan 608-737, Korea; E-Mails: venkatjchem@pknu.ac.kr (J.V.); manimaribtech@gmail.com (P.M.); khkang@pknu.ac.kr (K.-H.K.); 2 Department of Marine-bio Convergence Science, Pukyong National University, Busan 608-737, Korea; E-Mail: babolowe@gmail.com; 3 Stem Cell Unit, Department of Anatomy, King Saud University, Riyadh 12372, Saudi Arabia; E-Mail: paulelna@gmail.com; 4 Dental Biomaterials Research Chair, King Saud University, Riyadh 12372, Saudi Arabia; E-Mail: drsanil@gmail.com; 5 Specialized Graduate School Science & Technology Convergence, Pukyong National University, Busan 608-739, Korea

**Keywords:** hydroxyapatite, salmon, fish, alkaline hydrolysis

## Abstract

Nano-Hydroxyapatite (nHA) was isolated from salmon bone by alkaline hydrolysis. The resulting nHA was characterized using several analytical tools, including thermogravimetric analysis (TGA), Fourier transform infrared spectroscopy (FT-IR), X-ray diffraction analysis (XRD), scanning electron microscopy (SEM) and transmission electron microscopy (TEM), to determine the purity of the nHA sample. The removal of organic matter from the raw fish was confirmed by TGA. FT-IR confirmed the presence of a carbonated group and the similarities to synthetic Sigma HA. XRD revealed that the isolated nHA was amorphous. Microscopy demonstrated that the isolated nHA possessed a nanostructure with a size range of 6–37 nm. The obtained nHA interacted with mesenchymal stem cells (MSCs) and was non-toxic. Increased mineralization was observed for nHA treated MSCs compared to the control group. These results suggest that nHA derived from salmon is a promising biomaterial in the field of bone tissue engineering.

## 1. Introduction

The increased need for organ replacement and repair is a worldwide human health challenge. The treatment of damaged tissue is typically performed using autologous and allogenic grafts. These methods are limited by insufficient donors and the high risk of disease transmission [1,2]. Significant achievements in the field of tissue engineering include artificial prostheses that can treat loss or failure or that can regenerate tissues and/or organs [3]. These advances would not have been realized without the contributions of biomaterials in the form of scaffolds that meet the requirements for optimal tissue formation [4]. Currently, natural biomaterials play pivotal roles in the design and production of biocompatible prostheses, biomimetics, elucidating specific cell functions, allowing cell-cell interactions and the formation of organized matrices for tissue regeneration [5]. 

Biomaterials with properties similar to bone tissue have been continuously studied for use in bone tissue engineering [6,7,8,9,10]. The use of composites consisting of calcium phosphates and type I collagen is one favorable approach to mimic the extracellular matrix of bone tissue [11]. Hydroxyapatite ceramics (HA, Ca_10_(PO_4_)_6_(OH)_2_) are biocompatible, and their bioactivity can strengthen bone-bond formation with other tissues through an osteoconductive mechanism [12]. Nano-Hydroxyapatite (nHA) can be produced in different ways from synthetic and natural sources. Synthetic methods used to fabricate nHA include precipitation [13,14], radio frequency thermal plasma [15], reverse micro emulsion [16], an emulsion liquid membrane system [17], the sol gel method [14] and hydrothermal methods [18]. However, the synthetic production of nHA often requires the use of hazardous chemicals, ageing processes and an imbalanced stoichiometric ratio. HA can also be isolated from bovine sources [19,20,21,22,23,24,25], fish scales [26,27,28], fish bone [29,30,31,32,33,34,35,36,37], *Lates calcarifer* [28], cuttlefish bone [38], cat fish bone [38,39] and cod fish bone [40]. Bovine and pork origins are often associated with disease transmission and religious sentiments [41]. Fish sources are presumably much safer, and the wide evolutionary gap between fish and humans suggests a low risk of disease transmission [11]. Additionally, fish byproducts are abundant, and an application for these byproducts suitable for biomedical application would reduce environmental pollution and the threats of biohazards to humans. Mesenchymal stem cells (MSCs) have the capacity to differentiate into osteoblasts, chondrocytes, adipocytes, muscle cells and nerve cells *in vitro* and *in vivo*. They are also studied as a common cell source for bone tissue engineering applications [42,43]. 

We developed nHA from salmon fish bone through alkaline hydrolysis to mimic the extracellular matrix of bone. Thus, we have isolated nHA from salmon fish bone. Carbonated nHA was produced using the alkaline hydrolysis method. Characterization of the isolated nHA revealed that it is amorphous with nanoparticle sizes that range from 6–37 nm. We investigated the interaction between MSCs and nHA from salmon bone. The results demonstrated an increased biomineralization, possibly induced by the nHA, thus elucidating the differentiation capacity of MSCs into osteoblasts producing cells. This work suggests that nHA from salmon fish bone is an excellent biomaterial candidate for bone tissue engineering applications.

## 2. Results and Discussion

### 2.1. General Observations

Salmon fish are abundant in South Korea and are widely used as a food source. Salmon waste (scales, skin and bones) is discarded by local fish markets and industrial companies. This waste is hazardous to the ecosystem and represents an environmental and health risk. To avert this risk, we employed a cost efficient method of producing nHA from salmon bones for commercial and biomedical applications. Initially, raw salmon bone is covered by fish tissue and stored in a freezer. Crushed salmon bone is yellow in color. After alkali treatment, a light yellow color was observed. This color change indicates the removal of organic matter from the crushed bone. 

### 2.2. Thermogravimetric Results 

**Figure 1:**
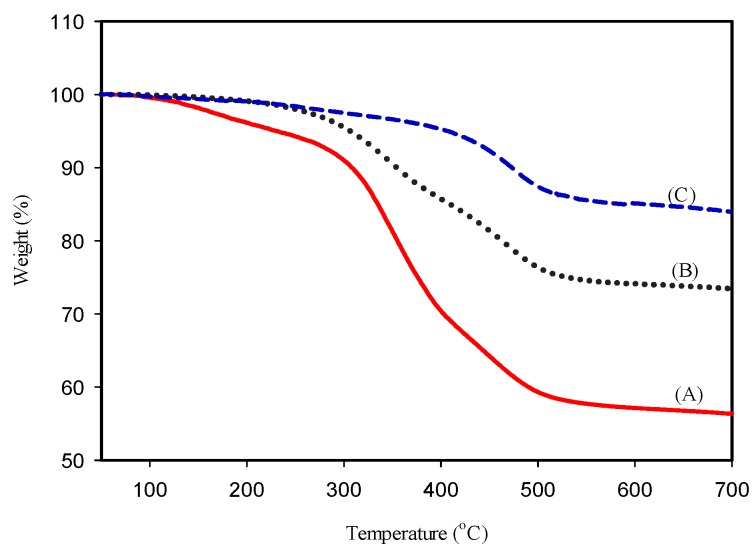
Thermogravimetric analysis of (**A**) raw salmon bone, (**B**) crushed salmon bone and (**C**) Nano-Hydroxyapatite (nHA) salmon bone after alkaline treatment.

The thermogravimetric analyses (50–700 °C) of raw bone, crushed bone and nHA-salmon are depicted in Figure 1. Two different points of weight loss (350 and 463 °C) were observed in the TGA spectrum of raw bone and crushed bone. These temperatures correspond to the organic moieties. Alkaline treated crushed bone demonstrates only one deflection at 465 °C, which may be attributed to the small amount of organic moieties present in the HA salmon bone. 

### 2.3. FT-IR Spectra Results

**Figure 2:**
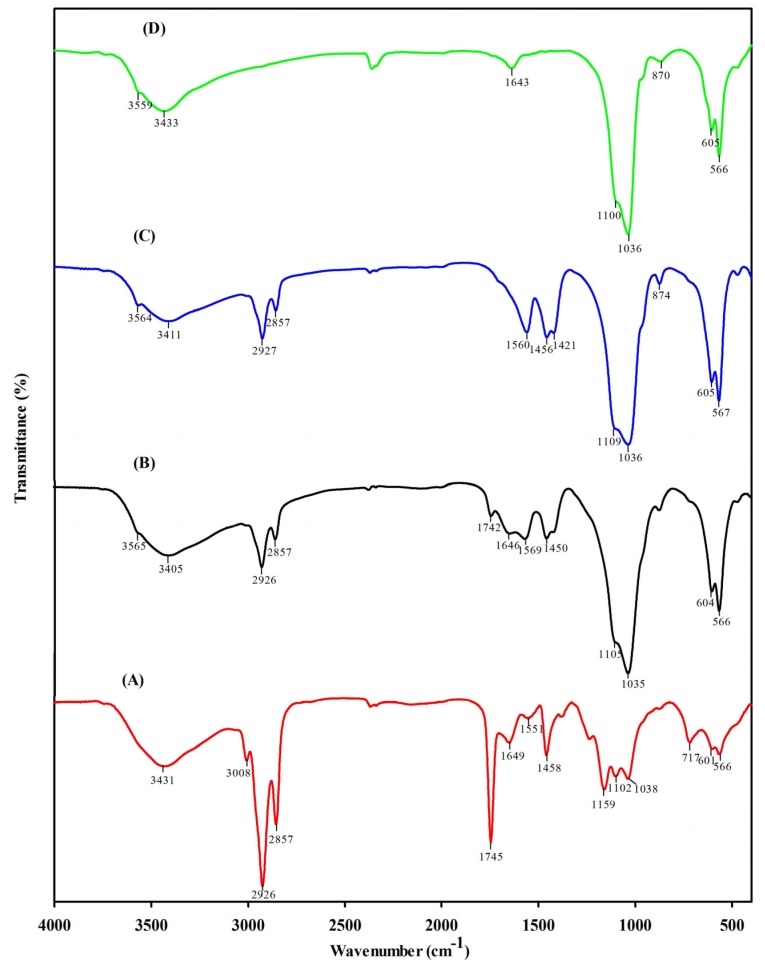
The fourier transform infrared spectroscopy (FT-IR) spectra of (**A**) raw salmon bone; (**B**) crushed salmon bone; (**C**) nHA salmon bone after alkaline treatment and (**D**) HA Sigma.

Fourier transform infrared spectroscopy (FT-IR) is a reliable reference technique to study the intra- and intermolecular interactions of a material. FT-IR was performed to identity the functional group of the isolated nHA. Figure 2A depicts the FT-IR spectrum of raw bone, crushed bone, nHA-salmon and synthetic HA from Sigma. The characteristic bands of raw bone were observed at 566, 601, 717, 1038, 1102, 1159, 1458, 1551, 1649, 1745, 2857, 2926, 3008 and 3431 cm^−1^. These bands indicate calcium phosphate and collagen moieties. A strong band was observed at 1000–1100 cm^−1^, indicating the stretching mode of PO_4_ vibration. A band at 567 cm^−1^ corresponds to the n4 symmetric P–O stretching vibration of a PO_4_ group. The band at approximately 3400 cm^−1^ corresponds to the O–H stretching of nHA [27]. Figure 2B depicts the crushed bone IR spectra. The 1745 cm^−1^ band was reduced, suggesting the removal of organic matter. However, the organic matter remains, as indicated by bands at 1450, 1569, 1646 and 1742 cm^−1^. These bands correspond to collagen. Figure 2C represents the alkaline hydrolysis derived nHA. Significant differences were observed for the stretching frequencies of nHA salmon compared to those of raw bone and crushed bone. Several bands that were present in crushed bone were absent in nHA salmon, indicating the removal of organic matter from the crushed bone. The characteristic bands of nHA salmon are 567, 605, 874, 1036, 1109, 1421, 1456, 1560, 2857, 2827, 3411 and 3564 cm^−1^. The bands of the carbonated group (A and B type) are 1560, 1421 and 1456 cm^−1^. Figure 2D depicts the infrared spectrum of HA from Sigma-Aldrich (St. Louis, MO, USA). The characteristic bands are 566, 605, 870, 1036, 1100, 1643, 3433 and 3559 cm^−1^. Differences were observed between HA Sigma and nHA salmon. Carbonated groups are absent in the HA Sigma.

### 2.4. X-Ray Diffraction Results

**Figure 3:**
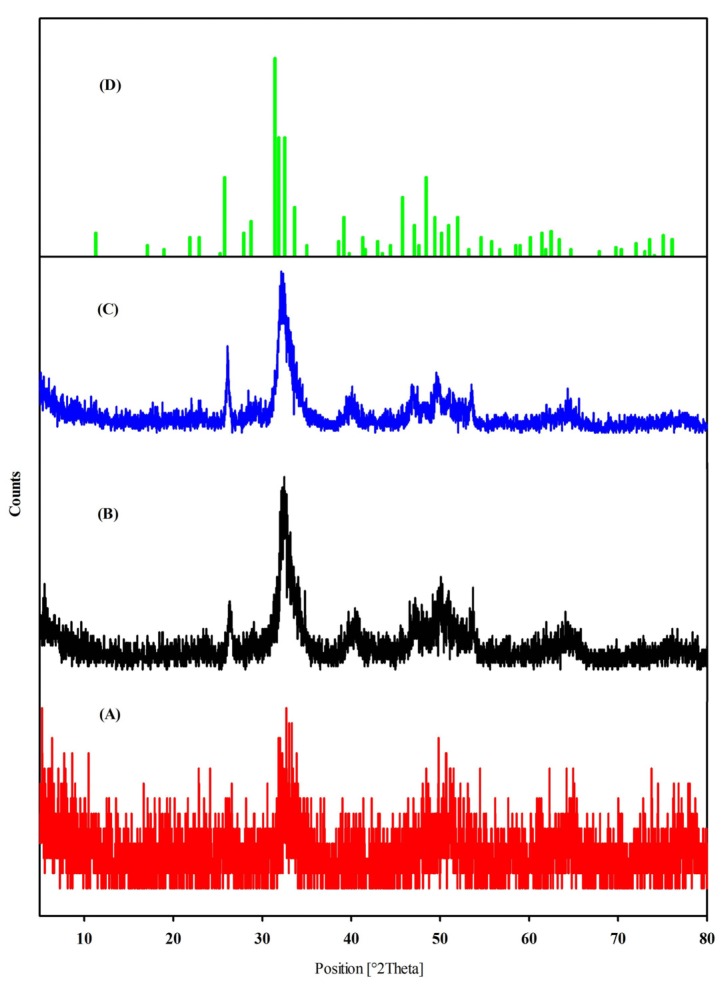
X-ray diffraction spectra of (**A**) raw salmon bone, (**B**) crushed salmon bone and (**C**) nHA salmon bone after alkaline treatment and (**D**) JCPDS 090432.

A broad single peak was observed in the X-Ray diffraction spectrum of raw bone at 32.7, confirming that the nHA is amorphous. In Figure 3B,C, two peaks were observed at 32.1 (211) and 26.3 (002) for crushed bone; 31.9 (211) and 26.1 (002) for nHA salmon bone. The intensities of the peaks were higher in crushed and HA salmon bone compared to raw bone. The highest intensity of the peaks of nHA (31.9) is similar to that of the standard JCPDS 090432 (31.7). X-ray diffraction analysis (XRD) analysis suggests that the purity of the nHA salmon is higher than that of crushed bone. 

### 2.5. Microscopy Results 

**Figure 4:**
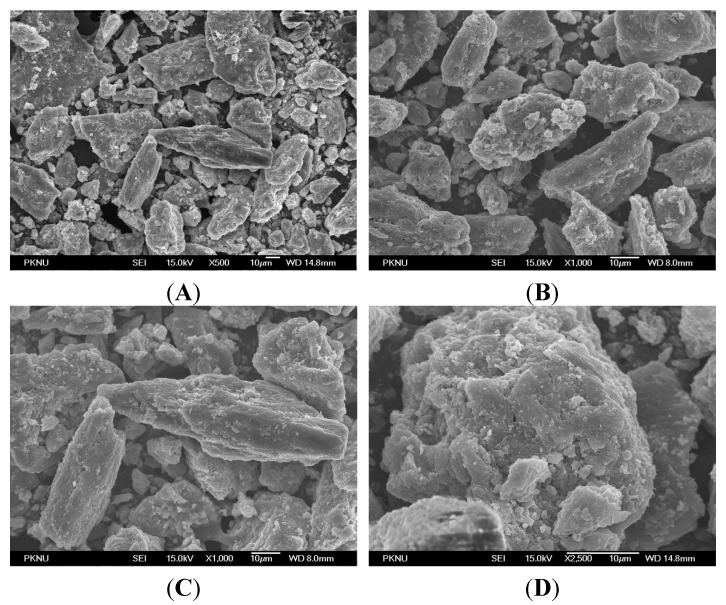
Field emission-scanning microscopy images of nHA salmon at different magnifications. (**A**) ×500; (**B**,**C**) ×1000 and (**D**) ×2500.

**Figure 5:**
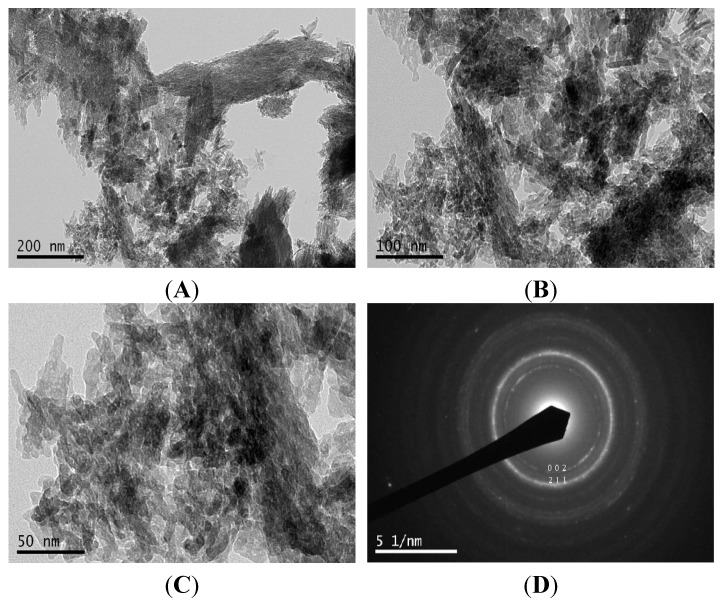
High Resolution Transmission Electron Microscopy (HR-TEM) micrographs demonstrating the appearance of the obtained nHA crystals at different scale bars: (**A**) 200 nm; (**B**) 100 nm and (**C**) 50 nm from fish bones after alkali treatment; (**D**) The corresponding selective area diffraction data of nHA.

Figure 4 depicts the FE-SEM images of nHA salmon at different magnifications: (**A**) ×500, (**B**,**C**) ×1000 and (**D**) ×2500. Agglomeration of the nHA particles was observed. Figure 5 depicts the transmission electron microscopy images of nHA salmon for different scale bars, (**A**) 200 nm, (**B**) 100 nm and (**C**) 50 nm, and (**D**) selective area image diffraction. TEM analysis demonstrated that the crystal sizes were 6–37 nm with a nanorod shape. The nHA salmon particles were also analyzed using selective area diffraction analysis. The selective area diffraction results were consistent with the XRD results with the planes (002) and (211). These studies also confirmed that the alkaline treatment did not affect the crystal size of HA salmon.

### 2.6. Cell Culture Results

**Figure 6:**
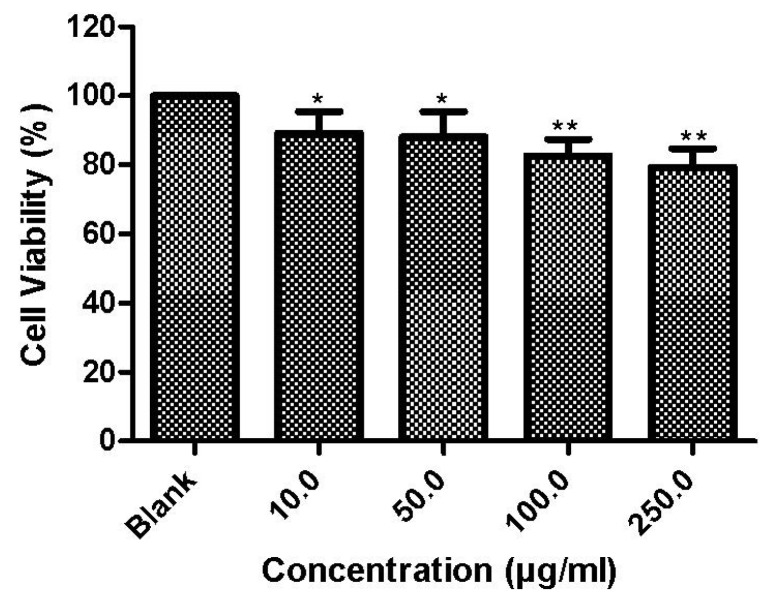
Cytotoxicity of nHA salmon crystals to mesenchymal stem cells at different concentrations. *: *P* ≤ 0.05; **: *P* ≤ 0.01.

3-(4,5-Dimethylthiazol-2-yl)-2,5-diphenyltetrazolium bromide (MTT) assay was used to study the cytotoxicity of nHA salmon crystals to MSCs. Figure 6 reports the cytotoxicity results of nHA with MSCs at different concentrations (10, 50, 100 and 250 μg/mL). The results suggest that nHA crystals are not toxic to cells at 100 μg/mL. The cell morphology of nHA treated MSCs was determined using optical microscopy. The results indicated a slight inhibition of cells at concentrations higher than 250 μg/mL. However, the cells proliferated without any toxic effect at lower concentrations. The cell growth of the control was similar to that of the individually seeded lower concentrations of nHA.

### 2.7. Morphological Results and Optical Microscopy

**Figure 7:**
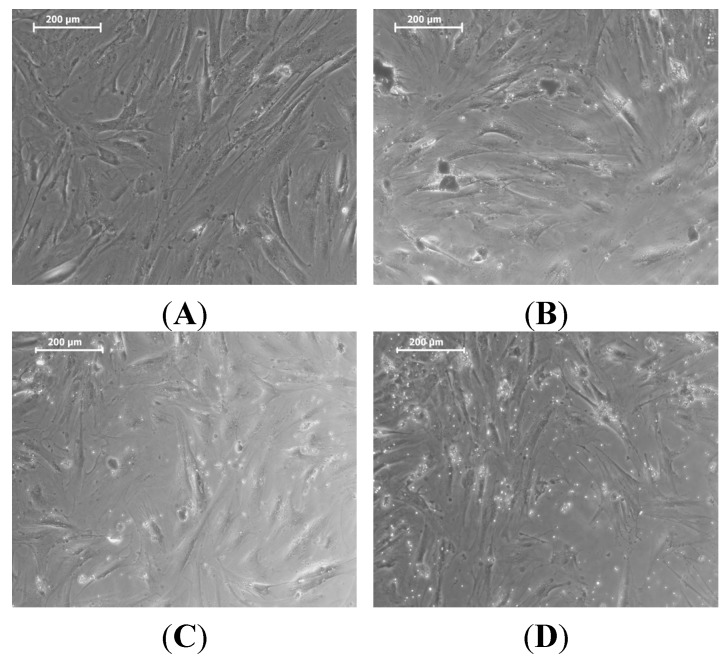
Phase contrast optical microscopy images of mesenchymal stem cells (MSCs) with nHA concentrations of (**A**) blank; (**B**) 50 μg/mL; (**C**) 100 μg/mL and (**D**) 250 μg/mL.

The morphology of the MSCs with nHA treatment was studied using optical microscopy (Figure 7). We observed no change in the morphologies of the MSCs with nHA. 

### 2.8. Mineralization Results

**Figure 8:**
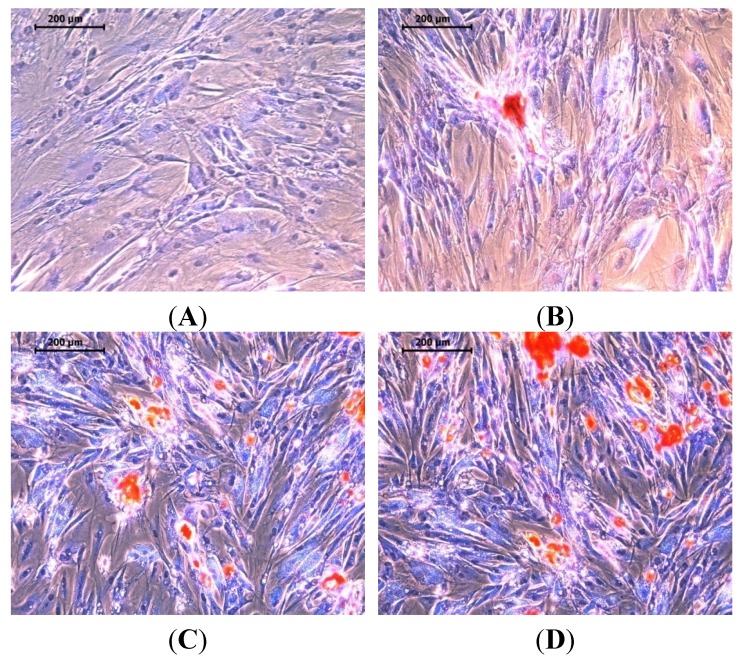
Alizarin red S stained images after 14 days of MSCs with nHA at 100 μg/mL for (**A**) Dulbecco’s Modified Eagle’s Medium (DMEM); (**B**) osteogenic differentiation medium and (**C**,**D**) 100 μg/mL nHA.

Studying mineralization is an important aspect in the development of advanced materials for use in organ regeneration. It also allows an understanding of how minerals can be produced by tissues [44]. The extracellular matrix is a highly organized nanocomposite that models cell function and biochemical processes. Tissue engineers use a nanotechnological approach to mimic this important matrix [45]. Binding sites exists in the form of nano-scaled fibers for cell adhesion. Nano-scaled protein fibers provide elasticity and strength [46]. The most stable form of calcium phosphate is nHA. It forms 70% of the dynamic and highly vascularized bone tissue. Figure 8 depicts the mineralization effect of nHA with MSCs for (**A**) Dulbecco’s Modified Eagle’s Medium (DMEM), (**B**) osteogenic differentiation medium (ODM) and (**C**,**D**) 100 μg/mL nHA salmon treated with ODM media. These results demonstrate the production of minerals by MSCs induced by the nHA salmon. Quantification of the minerals produced by the control, ODM and HA treatments showed relative mineralization percentage of 100%, 104% and 118.26%, respectively. This result implies that the HA salmon crystals induce mineralization. 

## 3. Experimental Section 

### 3.1. Preparation of Salmon Fish Bone

Salmon fish bones were supplied from a local fish market, Busan, South Korea. Bones were cut into smaller pieces using a wooden hammer and a bladed cutter. Bones were boiled with 2 L H_2_O at 200 °C for 3 h to remove the flesh. 1 L H_2_O was further added and boiled for 4 h to remove all tissue remnants. Washed bones were collected and further boiled with 10 mL of acetone and 2% NaOH (10 g/500 mL of H_2_O) for 1 h to remove the remaining tissue. The solution was continually flushed with H_2_O to ensure tissue removal and was oven dried at 100 °C for 3 h to remove moisture. The bones were crushed with a mortar and pestle. 

### 3.2. Isolation of Hydroxyapatite from Salmon Bone 

nHA was isolated from salmon bone using alkaline hydrolysis [35]. Crushed bone (10 g) was heated with 2 M NaOH (Junsei Chemical Co., Ltd., Tokyo, Japan) for 1 h at 200 °C. This process was repeated until all traces of organic and collagenous material were removed. nHA was collected into conical tubes, centrifuged (Combi-514R, Hanil Science Industrial Co., Ltd., Incheon, Korea) at 1000 rpm for 5 min, washed with H_2_O until it reached neutral pH and dried in an oven at 100 °C.

### 3.3. Chemical Characterization Methods

#### 3.3.1. Thermogravimetric Analysis

Thermogravimetric analyses of nHA were performed using a Pyris 1 TGA analyzer (Perkin-Elmer TGA-7, Waltham, MA, USA) with a scan range from 50 to 700 °C and a constant heating rate of 10 °C·min^−1^ under continuous nitrogen.

#### 3.3.2. Fourier Transform Infrared Spectroscopy

Infrared spectrum resolution frequencies of the nHA were determined by Fourier transform infrared spectroscopy (JASCO FT/IR-4100, JASCO, Tokyo, Japan) and a spectra manager (Serial number: C251761016) with a range of 400 to 4000 cm^−1^.

#### 3.3.3. X-Ray Diffraction Analysis

The atomic and molecular structure of the nHA crystals were analyzed using an X-ray diffractometer (PHILIPS X’pert MPD, PANalytical, Almelo, The Netherland); Cu-Kα radiation (1.5405 Å) over a range of 5° to 80°, a step size of 0.02 and a scan speed of 4°·min^−1^ at 40 kV and 30 mA were used.

#### 3.3.4. Microscopic Analyses

The morphology of the nHA crystals was characterized by field-emission scanning electron microscopy (FESEM, JSM-6700F, JEOL, Tokyo, Japan) and transmission electron microscopy (HITACHIH-7500, Hitachi, Ltd., Tokyo, Japan). 

#### 3.3.5. Cell Culture Studies 

MSCs were purchased from ATCC (American Tissue Culture Collection, Manassas, VA, USA) and were cultured in Dulbecco’s Modified Eagle’s Medium (BioWhittaker^®^, Madison, WI, USA) containing 10% Fetal bovine serum (FBS) (Serana^®^, Bunbury, Australia) and 10 mL Penicillin-Streptomycin (BioWhittaker^®^) in a 37 °C humidified atmosphere of 5% CO_2_.

#### 3.3.6. Cytotoxicity Assessment 

MSCs were cultured in a cell culture dish 100 × 200 mm (SPL life sciences, Gyeonggi-do, Korea) in a humidified incubator (SANYO CO_2_ Incubator-MCO-15AC, SANYO Electric Co., Ltd., Osaka, Japan) of 5% CO_2_ at 37 °C containing 10% FBS and 10 mL antibodies (Penicillin-Streptomycin). Cells were harvested when confluent and then seeded in a 24-well plate containing 1 mL medium at a final density of 1 × 10^5^ cells/mL. Concentrations (250, 100, 50, 10 and 0 μg/mL (blank)) of isolated nHA were added to each plate. Cells were incubated for 24 h. The media were removed, and 1 mL of MTT (0.0125 mg/25 mL) was added to each well and incubated for 4 h at 37 °C. The MTT was removed, and formazan crystals were stabilized by the addition of Dimethyl sulfoxide (DMSO) (1 mL/well). The MTT assay was quantified using a GENios^®^ microplate reader (Tecan Austria GmBH, Grödig, Austria) at an absorbance of 570 nm. 

#### 3.3.7. Optical Microscopy

To examine the interaction of nHA and MSCs, cells were fixed with 2.5% glutaraldehyde (Sigma-Aldrich). Cells were examined using an optical microscope (CTR 600; Leica, Wetzlar, Germany).

#### 3.3.8. Mineralization Assay

The amount of mineralization produced was quantified by Alizarin Red-S (ARS) stain. DMEM medium, ODM media and nHA treated MSCs were incubated for 14 days. Cells were washed and treated with Alizarin Red-S (Sigma-Aldrich) (0.2 g/20 mL of H_2_O). The cells were fixed with 70% ethanol at room temperature for 1 h. The ethanol was removed, and 1 mL of Alizarin Red S (pH 4.2) was added for 15 min. Cells were washed with H_2_O, and the optical microscopy (CTR 6000; Leica, Wetzlar, Germany) images were taken. To quantify the minerals, 1 g of cetylpyridinium chloride (Wako Pure Chemical Industries Ltd., Osaka, Japan) was prepared with (0.2 g/20 mL of H_2_O) sodium phosphate (Sigma-Aldrich). Each well was treated with 1 mL of the prepared concentration for 15 min. The optical density was determined by a microplate reader (Tecan Austria GmBH, Grödig, Austria) at 562 nm.

#### 3.3.9. Statistical Analyses

Statistical analyses were performed by Graphpad Prism 5. All experiments were run in triplicate, and the data were presented as the mean value ± standard deviation (SD) of each group.

## 4. Conclusions

We have isolated pure nHA from salmon fish bone using the alkaline hydrolysis method. This is a cost efficient method for the isolation of nHA. FT-IR results confirmed the presence of a carbonated group (preferable for biomedical applications). XRD results determined that the crystals are amorphous. Scanning electron and transmission electron microscopy results revealed that the crystals exhibit a nanostructure with a size range of 6–37 nm. Cytotoxicity analysis of isolated nHA and MSCs suggests that the nHA is nontoxic and biocompatible. The nHA induced higher mineralization in the MSCs, which is important for bone tissue engineering applications. The successful isolation and characterization of this important nano-material will be useful in biomedical applications, especially in bone tissue engineering through its development. This material may reduce the environmental effect of byproducts from the salmon industry while efficiently safeguarding industrial pollution and waste management. This research suggests that nHA salmon is an alternative biomaterial with potential for biomedical applications in the field of bone tissue engineering. 
